# Lower extremity function and cardiovascular disease risk in hemodialysis patients: A multicenter cross‐sectional study

**DOI:** 10.14814/phy2.70014

**Published:** 2024-08-20

**Authors:** Kun Zhang, Xin Li, Qi Guo, Wei Ding, Jianying Niu, Junli Zhao, Liming Zhang, Hualin Qi, Suhua Zhang, Chen Yu

**Affiliations:** ^1^ Department of Nephrology, Tongji Hospital, School of Medicine Tongji University Shanghai China; ^2^ Department of Rehabilitation Medicine Shanghai University of Medicine and Health Sciences Affiliated Zhoupu Hospital Shanghai China; ^3^ Department of Nephrology, Shanghai Ninth People's Hospital Shanghai Jiao Tong University School of Medicine Shanghai China; ^4^ Department of Nephrology, The Fifth People's Hospital of Shanghai Fudan University Shanghai China; ^5^ Department of Nephrology Shanghai University of Medicine and Health Sciences Affiliated Zhoupu Hospital Shanghai China; ^6^ Department of Nephrology Zhabei Central Hospital of JingAn District of Shanghai Shanghai China; ^7^ Department of Nephrology Shanghai Pudong New Area People's Hospital Shanghai China; ^8^ Department of Nephrology Suzhou Kowloon Hospital, Shanghai Jiaotong University School of Medicine Shanghai China

**Keywords:** cardiovascular disease, hemodialysis, lower extremity function

## Abstract

Physical performance in hemodialysis patients declines and serves as a cardiovascular disease (CVD) incidence and mortality predictor. However, lower extremity function's role remains unclear. This study aimed to quantify the association between lower extremity function and CVD risk in hemodialysis patients. This was a multicenter cross‐sectional study enrolling 868 participants (532 males, 336 females) from seven hemodialysis centers in Shanghai, China. Patients were divided into three groups per lower extremity function, evaluated by short physical performance battery (SPPB) scores: 0–6, 7–9, and 10–12. Upper extremity function was quantified through grip strength assessment. CVD risk was assessed using the Framingham Risk Score. Approximately 35% of hemodialysis patients had impaired lower extremity function (SPPB score < 10). Participants with high SPPB scores had stronger handgrip and lower Framingham CVD risk scores than those with low and moderate SPPB scores (*p* < 0.05). After adjusting clinical confounders, SPPB was independently associated with CVD risk, as a categorized variable (odds ratio: 0.577, 95% confidence interval [CI]: 0.388–0.857, *p* = 0.006) and as a continuous variable (odds ratio: 0.858, 95% CI: 0.772–0.953, *p* = 0.004). An SPPB score < 10 predicted an increased CVD risk (area under curve: 0.649, 95% CI: 0.599–0.699, *p* < 0.001). Causality between physical performance and CVD risk was not considered. Some upper limb results may not be generalizable to peritoneal dialysis and kidney transplant patients. Lower extremity function was significantly associated with CVD risk in hemodialysis patients. Further studies are needed to explore the long‐term relationship between lower extremity function and CVD risk.

## 1 INTRODUCTION

Cardiovascular disease (CVD) is an increasingly serious problem as an autonomous predictor of mortality in patients undergoing maintenance hemodialysis (MHD) (Cozzolino et al., 2018; Tong et al., 2016). The US Renal Data System 2021 revealed that the prevalence of CVD in patients receiving MHD was 77.3%, with over half of known deaths being related to CVD (Johansen et al., 2022). Furthermore, CVD‐related mortality is 10–30 times higher among patients on MHD than that in the common population (Herzog et al., 2011). Therefore, early assessment of atherosclerotic CVD risk in such patients is essential. The Framingham Risk Score (FRS) has been extensively used to predict the 10‐year risk of CVD events (D'Agostino Sr et al., 2008) and is adjusted as a confounder in analyses involving patients with chronic kidney disease (CKD) (Weiner et al., 2007).

Physical performance significantly declines in patients on MHD and is associated with undesirable outcomes, such as CVD‐related mortality (Torino et al., 2014). Components of physical performance include upper and lower extremity functions, which are strongly associated with CVD‐related mortality and affect the prediction of CVD risk (Welsh et al., 2020; Yates et al., 2017). However, upper and lower extremity functions play different roles in cardiovascular function. For example, lower extremity function can contribute to preload and cardiac output (Halkar et al., 2020), whereas handgrip and endothelial function are closely associated with the vascular system (Yoo et al., 2018). Furthermore, compared with handgrip strength, a study confirmed that a slow walking speed showed a higher risk for all‐cause and CVD‐related mortalities in the general population through multi‐level adjustment (Yates et al., 2017). However, the correlation between lower extremity function and CVD risk in patients on MHD remains unclear.

In this analysis, we aimed to explore the predictive value of lower extremity physical performance, which was assessed using the short physical performance battery (SPPB), and CVD risk in a multicenter cohort of patients on MHD.

## 2 MATERIALS AND METHODS

### 2.1 Participants

This cross‐sectional study included patients who underwent MHD at seven hemodialysis units in Shanghai, China, between July 2020 and April 2021 (ChiCTR1900027039). The inclusion criteria were as follows: patients (1) aged >18 years and (2) on hemodialysis for more than 3 months. The exclusion criteria were as follows: (1) a lack of laboratory measurements; (2) a combination with peritoneal dialysis treatment; (3) any incomplete physical tests; and (4) any signs of overt clinical infection, acute CVD, or malignancy. The study was authorized by the Ethics Committee of Tongji Hospital, Tongji University (K‐2020‐024), and the study was carried out adhering to the principles of the Declaration of Helsinki. Prior to enrollment in the study, informed consent was obtained from all participants in written form.

### 2.2 Baseline variables and laboratory measurements

Structured questionnaires were used for data collection and were obtained through face‐to‐face interviews. The questionnaire comprised inquiries pertaining to participants' demographic characteristics (age, sex, dry body weight, and height), medical history (dialysis vintage, diabetes, hypertension, or other diseases), and lifestyle habits (smoking and drinking habits). The body mass index (BMI) was calculated as the weight (kg) divided by the square of the height (m) (Tong et al., 2016). Hemoglobin, albumin, calcium, phosphate, intact parathyroid hormone (iPTH), urea, total cholesterol, and low‐density lipoprotein levels were measured using standard laboratory techniques. Moreover, every blood sample was drawn before dialysis. The fractional clearance index for urea (Kt/V) was calculated using single‐pool urea kinetic modeling (Gotch, 2001).

### 2.3 Muscle function

Muscle strength and physical performance were assessed for muscle function. Handgrip, which represents upper limb muscle strength, was measured in the non‐fistula limb if the fistula was implanted or in the dominant hand if a tunnel‐cuffed catheter was inserted in the neck, using a dynamometer (GRIP–D; Takei Ltd, Niigata, Japan). Handgrip testing was conducted during the interdialytic period, with participants positioned in a standing posture. The maximum effort of participants was recorded twice, and data on the strongest handgrip strength were analyzed.

Lower extremity physical function was measured using the SPPB scores developed by the National Institute on aging research fellows (Guralnik et al., 1994). The SPPB is a performance‐based method consisting of three tests: standing balance, walking pace, and chair stand. Each test required participants to stand on their feet for 10 seconds, with progressively more difficult stances: feet side by side, semi–tandem, and full tandem. Walking speed was assessed using the 4–m walk test at the usual velocity, with or without a gait‐assistance device. In the chair stand test, participants were asked to stand unassisted from a chair and sit on it rapidly with their upper limbs folded on the chest. This was repeated five times. Each item was rated from 0 to 4, with a total score between 0 and 12 and lower scores representing a poorer performance. The degree of reliability of the SPPB in this population is considered acceptable (Ortega–Pérez et al., 2018). Based on previously defined categories, the participants were classified into the following groups based on their SPPB score range: low (SPPB score range = 0–6), moderate (7–9), and high (10–12) (Bellettiere et al., 2009).

### 2.4 Assessment of CVD risk

The 10‐year CVD risk was estimated according to the FRS (Cedeño Mora et al., 2017; D'Agostino Sr et al., 2008; Expert Panel On Detection, Evaluation, and Treatment of High Blood Cholesterol in Adults, 2001). The FRS was calculated based on age, sex, systolic blood pressure, total cholesterol, high‐density lipoprotein cholesterol, hypertension, diabetes mellitus, and smoking history. Moreover, within the FRS algorithm, every risk factor was assigned according to sex, and a summary FRS was translated into the CVD risk ratio. The cutoff point for high cardiovascular risk was an FRS of 10%.

### 2.5 Charlson Comorbidity Index and malnutrition inflammation score

The Charlson Comorbidity Index (CCI) is used to estimate comorbid conditions among patients with end‐stage renal disease (ESRD) by creating a sum score of 19 comorbid conditions (Liu et al., 2010). The CCI has also been used to describe comorbidity burden and predict outcomes (Liu et al., 2021).

The malnutrition inflammation score (MIS) is a rating scale of nutrition for morbidity and mortality in dialysis patients (Gencer et al., 2019; Kalantar‐Zadeh et al., 2001). The MIS contains 10 components of the nutritional and functional state, and each component has four layers, from 0 (normal) to 3 (severe). Higher scores indicate a more severe malnutrition status.

### 2.6 Statistical analyses

Continuous data are expressed as means ± standard deviations for normally distributed variables or as medians (25th–75th percentile) for non‐normally distributed variables. Categorical data are expressed as frequencies (percentages). We analyzed participant characteristics according to SPPB categories using the analysis of variance, the Kruskal–Wallis test for continuous data, and the chi‐squared test for categorical data. Pearson's and Spearman's correlation coefficients were used to calculate the correlations between CVD risk and other variables. An FRS <10% and ≥10% were defined as low and medium‐to‐high CVD risk, respectively. Univariable and multivariable logistic regression models were used to identify the associations of sociodemographic factors, laboratory parameters, and physical performance with CVD risk. All analyses were performed with SPPB as both a continuous variable and categorized variable with SPPB groups. The crude model was unadjusted. Sex and BMI were adjusted in model 1. The dialysis vintage, smoking history, CCI, and MIS were added to the variables in model 2, and finally, Kt/V, albumin, calcium, phosphate, and iPTH were added to the variables in model 3 as adjusted variables. Receiver operating characteristic curve analysis was used to validate the area under the curve (AUC) and assess the significance of SPPB in predicting CVD risk. In addition, sensitivity and specificity analyses were performed to identify the optimal cutoff value for SPPB in predicting CVD risk. A discrepancy was considered significant at *p* < 0.05. Statistical analyses were performed using standard statistical software packages (SPSS Inc., version 26.0, Chicago, Illinois, USA).

## 3 RESULTS

### 3.1 Baseline characteristics and different clinical variables of SPPB groups

**FIGURE 1:**
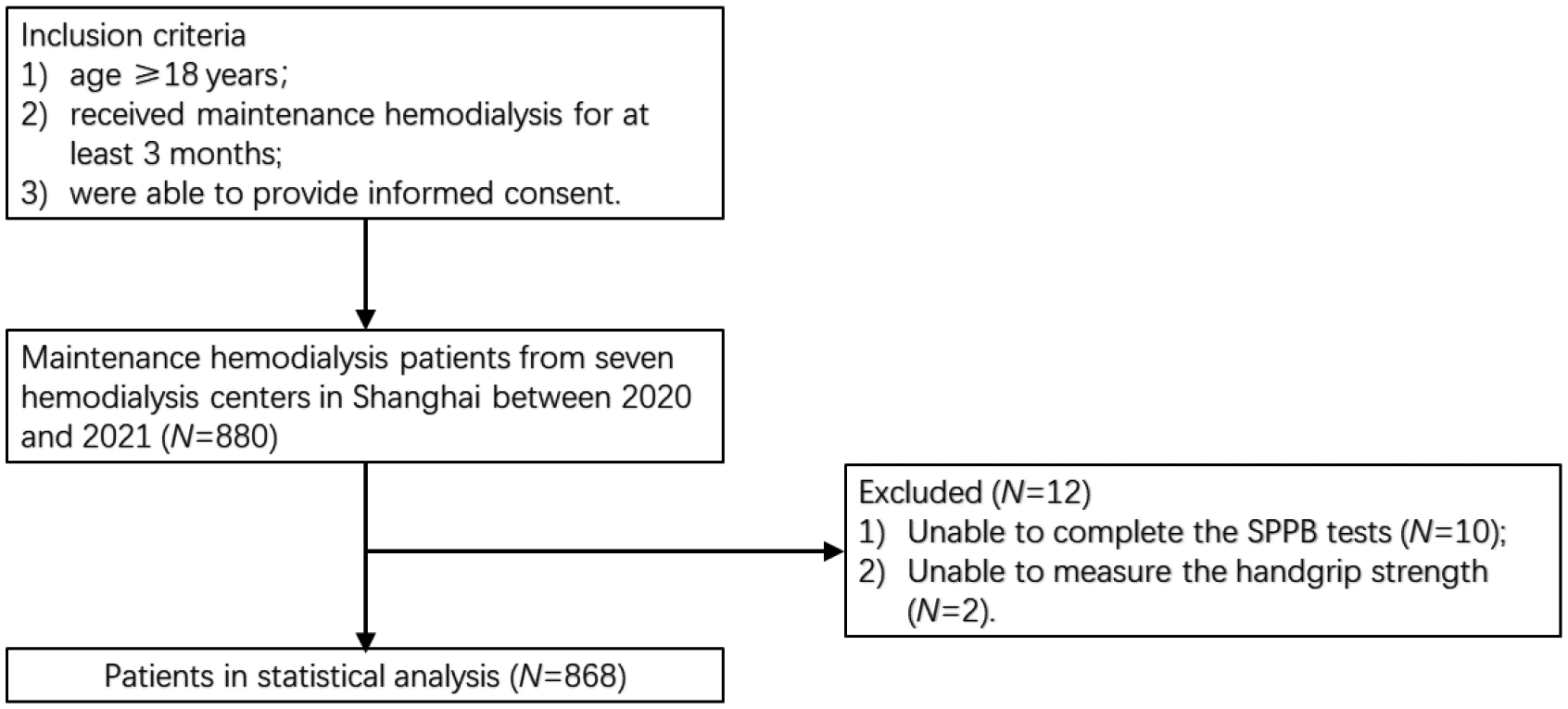
Flowchart of the study.

A total of 868 participants (532 males and 336 females; mean age: 61.5 ± 12.6 years) were enrolled in the study (shown in Figure 1). ESRD was caused by glomerulonephritis (*n* = 241, 27.8%), diabetes (*n* = 143, 16.5%), hypertension (*n* = 134, 15.4%), polycystic kidney disease (*n* = 71, 8.2%), and others (*n* = 279, 32.1%). The mean SPPB score was 9.6 ± 2.94 (out of 12), and the number of participants in the three SPPB groups were as follows: low, 131 patients (15.1%); moderate, 177 patients (20.4%); and high, 560 patients (64.5%). Participants with high SPPB scores were younger and had stronger handgrip strengths than those with low and moderate SPPB scores. Furthermore, the predicted 10‐year CVD risk using the FRS was 20.6% for participants in the high SPPB group, which was lower than those in the other two groups (low: 24.8% and moderate: 25.4%). Sex, BMI, drinking, dialysis vintage, serum albumin, iPTH, serum calcium, serum phosphate, blood lipid levels, and single‐pool Kt/V (spKt/V) were not significantly different among the three groups. The baseline characteristics of the participants are shown in Table 1.

**TABLE 1:** Participant baseline characteristics according to SPPB categories.

	Total (*n* = 868)	Low SPPB (*n* = 131)	Moderate SPPB (*n* = 177)	High SPPB (*n* = 560)	*p*‐value
SPPB score		<7	7–9	>9	
Sex (Male) *n* (%)	532	69 (52.6%)	109 (61.6%)	354 (63.2%)	0.083
Age (y)	61.5 ± 12.6	71.5 ± 11.2	65.8 ± 10.8	57.6 ± 12.7	<0.0001
BMI (kg/m^2^)	23.3 ± 3.8	23.71 ± 3.8	23.7 ± 3.7	23.3 ± 3.8	0.053
Smoking history *n* (%)	411	53 (40.5%)	89 (50.3%)	269 (48.0%)	0.192
Drinking history *n* (%)	96	9 (6.9%)	19 (10.7%)	68 (12.1%)	0.233
Dialysis Vintage (months)	65.0 ± 55.8	62.0 ± 55.9	58.7 ± 50.3	67.7 ± 57.3	0.144
Laboratory parameters					
Hemoglobin (g/dL)	110.9 ± 15.8	107.0 ± 15.6	111.0 ± 15.2	111.9 ± 15.9	<0.01
Albumin (g/L)	40.3 ± 3.4	38.1 ± 3.8	41.6 ± 3.1	40.4 ± 3.0	0.212
iPTH (pg/dL)	274.4 (143.3, 469.2)	234.0 (110.9, 432.7)	264.3 (131.6, 391.7)	282.4 (163.6, 495.9)	0.176
Calcium (mmol/L)	2.27 ± 0.25	2.22 ± 0.25	2.26 ± 0.27	2.27 ± 0.25	0.054
Phosphate (mmol/L)	1.96 ± 0.64	1.89 ± 0.76	1.92 ± 0.61	1.99 ± 0.62	0.196
TCH (mmol/L)	3.91 ± 1.20	3.79 ± 1.10	3.91 ± 1.20	3.94 ± 1.22	0.424
TG (mmol/L)	2.30 ± 0.8	2.21 ± 0.83	2.32 ± 0.89	2.31 ± 0.77	0.409
spKt/V	1.37 ± 0.33	1.38 ± 0.39	1.37 ± 0.35	1.37 ± 0.31	0.962
Physical performance					
SPPB total score	9.6 ± 2.94	3.82 ± 1.82	8.13 ± 0.81	11.41 ± 0.78	<0.001
Standing balance	3.05 ± 1.18	1.39 ± 1.11	2.30 ± 0.99	3.67 ± 0.62	<0.001
Gait speed (m/s)	0.98 ± 0.31	0.49 ± 0.19	0.86 ± 0.20	1.13 ± 0.20	<0.001
Chair stands (s)	11.1 ± 4.41	19.3 ± 6.80	13.6 ± 3.69	9.3 ± 2.02	<0.001
Handgrip (kg)	24.7 ± 8.8	17.3 ± 6.4	21.7 ± 6.9	27.6 ± 8.3	<0.001
Comorbidity					
CCI	3.87 ± 1.68	4.89 ± 1.91	4.42 ± 1.69	3.44 ± 1.43	<0.001
MIS	4.34 ± 2.88	6.46 ± 3.42	4.44 ± 2.84	3.75 ± 2.40	<0.001
FRS (%)	22.23 ± 9.22	24.8 ± 7.6	25.4 ± 7.4	20.6 ± 9.7	<0.001

Abbreviations: BMI, body mass index; CCI, Charlson Comorbidity Index; FRS, Framingham Risk Score; iPTH, intact parathyroid hormone; LDL–C, low‐density lipoprotein C; MIS, malnutrition inflammation score; spKt/V, single‐pool fractional clearance index for urea; SPPB, short physical performance battery; TCH, total cholesterol.

### 3.2 Association between clinical variables

We explored the association between the FRS for CVD, clinical variables, and physical function in patients with MHD, as shown in Table 2. Correlations between patient characteristics and the FRS for CVD showed that the data of demographics, comorbidities (age, sex, BMI, dialysis vintage, and CCI), and clinical indicators (serum albumin, iPTH, calcium, phosphate, and spKt/V) were significantly associated with FRS for CVD (*p* < 0.05). The FRS for CVD was also correlated with SPPB and its three components (*p* < 0.01) but not with handgrip strength (*p* = 0.268).

**TABLE 2:** Association of the FRS with clinical variables and physical performance.

	Correlation	*p*‐value		Correlation	*p*‐value
Sex	−0.45	<0.001	Physical performance		
Age	0.546	<0.001	SPPB total score	−0.208	<0.001
BMI	0.123	<0.001	Standing balance	−0.23	<0.001
Dialysis vintage (months)	−0.234	<0.001	Gait speed (m/s)	−0.162	<0.001
Laboratory parameters			Chair stands (s)	0.182	<0.001
Hemoglobin (g/dL)	0.016	0.628	Handgrip (kg)	0.037	0.268
Albumin (g/L)	−0.073	0.03	Comorbidity		
iPTH (pg/dL)	−0.174	<0.001	CCI	0.314	<0.001
Calcium (mmol/L)	−0.079	0.019	MIS	0.033	0.327
Phosphate (mmol/L)	−0.083	0.015			
TCH (mmol/L)	0.035	0.296			
LDL–C (mmol/L)	0.037	0.279			
spKt/V	−0.117	0.001			

Abbreviations: BMI, body mass index; CCI, Charlson Comorbidity Index; FRS, Framingham Risk Score; iPTH, intact parathyroid hormone; LDL–C, low‐density lipoprotein C; MIS, malnutrition inflammation score; spKt/V, single‐pool fractional clearance index for urea; SPPB, short physical performance battery; TCH, total cholesterol.

### 3.3 Univariate and multivariate logistic regression analyses of predictors of FRS for CVD risk

Table 3 illustrates the associations between SPPB and FRS for CVD risk. In the crude model, the hazard ratios suggested that patients on hemodialysis with low SPPB had a significantly higher risk of CVD (odds ratio [OR]: 0.476, 95% confidence interval [CI]: 0.333–0.681, *p* < 0.001). This association remained statistically significant, even after adjusting for other clinical variables that may be associated with CVD risk in model 1 (OR: 0.440, 95% CI: 0.305–0.635, *p* < 0.001), model 2 (OR: 0.582, 95% CI: 0.394–0.859, *p* = 0.006), and model 3 (OR: 0.577, 95% CI: 0.388–0.857, *p* = 0.006) (Table 3). Similarly, when SPPB was analyzed as a continuous variable (OR: 0.858, 95% CI: 0.772–0.953, *p* = 0.004 in model 3), the results were consistent with those obtained from the analysis of SPPB as categorized variables.

**TABLE 3:** Univariate and multivariate logistic regression analyses predicting CVD risk in patients on hemodialysis.

	Categorized 95% CI	*p*‐value	Continuous 95% CI	*p*‐value
Crude	0.476 (0.333, 0.681)	<0.001	0.824 (0.751, 0.906)	<0.001
Model 1	0.440 (0.305, 0.635)	<0.001	0.809 (0.735, 0.890)	<0.001
Model 2	0.582 (0.394, 0.859)	0.006	0.863 (0.779, 0.957)	0.005
Model 3	0.577 (0.388,0.857)	0.006	0.858 (0.772,0.953)	0.004

*Note*: Reference for categorized: SPPB group. Crude, unadjusted; Model 1, adjusted for sex, BMI; Model 2, model 1 + dialysis vintage, smoking history, CCI, MIS; Model 3, model 2 + Kt/V, albumin, calcium, phosphate, iPTH.

Abbreviations: BMI, body mass index; CCI, Charlson Comorbidity Index; CI, confidence interval; HD, hemodialysis; iPTH, intact parathyroid hormone; MIS, malnutrition inflammation score; spKt/V, single‐pool fractional clearance index for urea; SPPB, short physical performance battery.

### 3.4 Subgroup analyses

Logistic regression models were utilized to examine the associations between SPPB as a continuous variable and CVD risk in various subgroups, with adjustments for the same covariates. The findings indicated that SPPB was significantly associated with CVD risk in several subgroups, including females (OR: 0.860, 95% CI: 0.758–0.976, *p* = 0.019), individuals with no smoking history (OR: 0.844, 95% CI: 0.749–0.951, *p* = 0.005), dialysis vintage less than 60 months (OR: 0.693, 95% CI: 0.560–0.858, *p* < 0.001), CCI scores both less or more than 4 (OR: 0.837, 95% CI: 0.724–0.969, *p* = 0.017; OR: 0.853, 95% CI: 0.730–0.996, *p* = 0.044, respectively), and MIS scores lower than 6 (OR: 0.784, 95% CI: 0.676–0.909, *p* = 0.001) (Table 4).

**TABLE 4:** Univariate and multivariate logistic regression analyses predicting CVD risk in patients on hemodialysis in subgroups.

	Model 1 (95% CI)	*P*‐value	Model 3 (95% CI)	*P*‐value
Sex				
Male	0.815 (0.684, 0.970)	0.021	0.909 (0.754, 1.097)	0.321
Female	0.807 (0.720, 0.905)	<0.001	0.860 (0.758, 0.976)	0.019
Smoking history				
No	0.799 (0.717, 0.890)	<0.001	0.844 (0.749, 0.951)	0.005
Yes	0.866 (0.699, 1.072)	0.186	0.946 (0.754, 1.186)	0.630
Vintage (months)				
<60	0.662 (0.545, 0.803)	<0.001	0.693 (0.560, 0.858)	<0.001
≥60	0.916 (0.817, 1.027)	0.131	0.974 (0.855, 1.110)	0.692
CCI				
CC <4	0.842 (0.733, 0.968)	0.016	0.837 (0.724, 0.969)	0.017
CCI ≥4	0.826 (0.719, 0.948)	0.007	0.853 (0.730, 0.996)	0.044
MIS				
MIS <6	0.775 (0.676, 0.888)	<0.001	0.784 (0.676, 0.909)	0.001
MIS ≥6	0.891 (0.774, 1.026)	0.109	0.984 (0.846, 1.144)	0.834

*Note*: SPPB as a continuous variable. Crude, unadjusted; Model 1, adjusted for sex, BMI; Model 2, model 1 + dialysis vintage, smoking history, CCI, MIS; Model 3, model 2 + Kt/V, albumin, calcium, phosphate, iPTH.

Abbreviations: BMI, body mass index; CCI, Charlson Comorbidity Index; CI, confidence interval; HD, hemodialysis; iPTH, intact parathyroid hormone; MIS, malnutrition inflammation score; spKt/V, single‐pool fractional clearance index for urea; SPPB, short physical performance battery.

### 3.5 Predictive ability of SPPB for CVD risks

**FIGURE 2:**
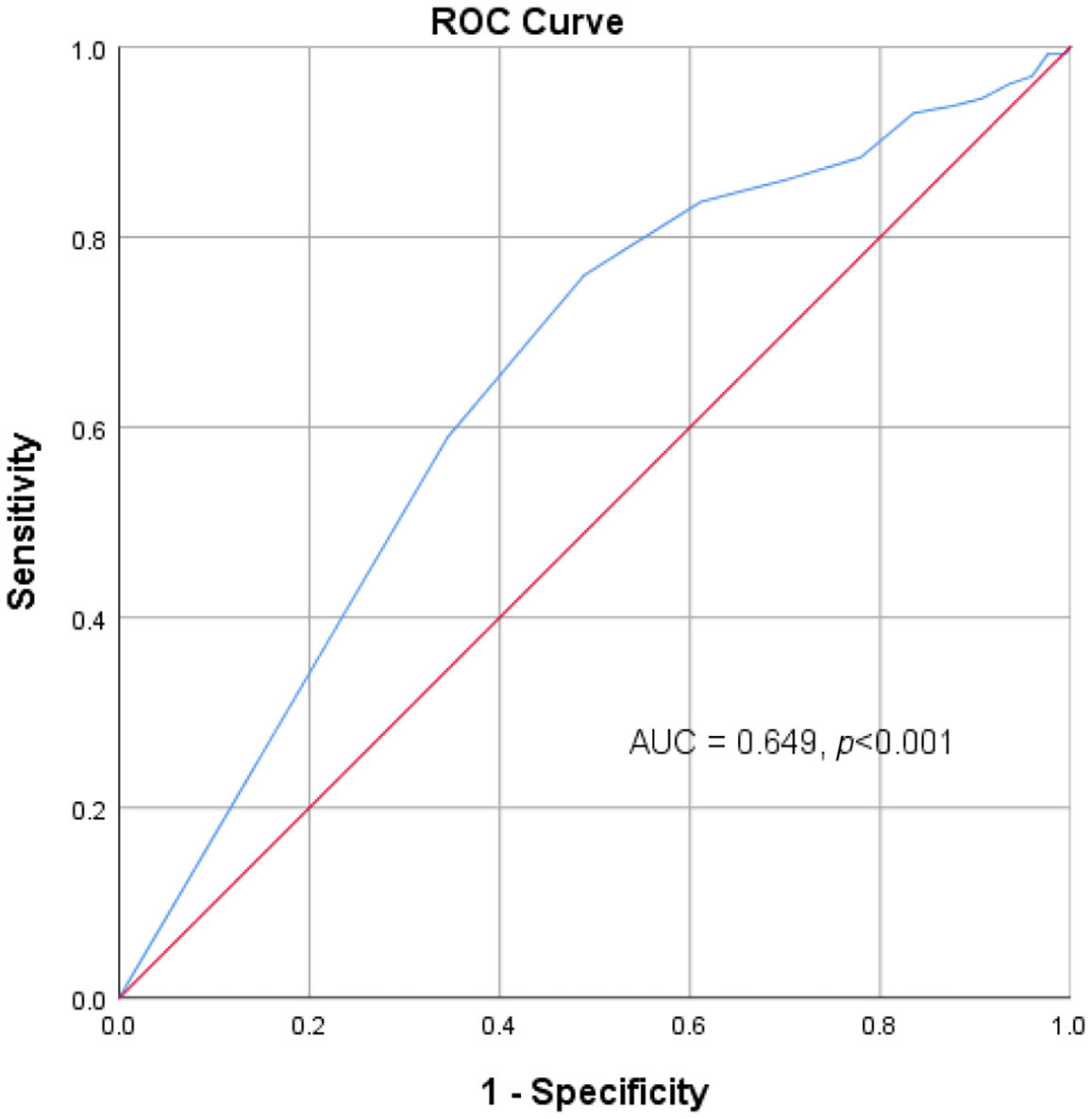
Predictive value of the SPPB for CVD risk. The ROC curve indicates the greatest AUC for the SPPB (0.649, 95% CI: 0.599–0.699; *p* < 0.001). AUC, area under the curve; CI, confidence interval; CVD, cardiovascular disease; ROC, receiver operating characteristic; SPPB, short physical performance battery.

To explore the predictive ability of the SPPB, we defined FRS <10% and ≥ 10% as low and medium‐to‐high CVD risk, respectively. The receiver operating characteristic curves indicated the greatest AUC for SPPB (0.649, 95% CI: 0.599–0.699; *p* < 0.001) (Figure 2). In comparison, handgrip strength (0.515, 95% CI: 0.5461–0.569; *p* = 0.591) had the lowest AUC for predicting CVD risk. Each component of the SPPB test also had a significant AUC for predicting CVD risk: standing balance (0.647, 95% CI: 0.598–0.695, *p* < 0.001), gait speed (0.606, 95% CI: 0.554–0.658, *p* = 0.04), and chair stand (0.593, 95% CI: 0.542–0.644, *p* = 0.001). The optimized cutoff point of the maximum predictive value calculated using the Youden's index for SPPB was 10.5, with a sensitivity of 0.766 and a specificity of 0.508. Therefore, a SPPB score of less than 10 can predict an increased CVD risk.

## 4 DISCUSSION

This study's results revealed that approximately 35.4% of patients undergoing hemodialysis had low and moderate SPPB scores (SPPB score <10) and a higher CVD risk than those with high SPPB scores. This indicated that the SPPB score had a strong inverse linear relationship with CVD risk. In addition, the SPPB components were also related to a higher risk of CVD. After adjusting for other clinical variables, the SPPB scores were still associated with CVD risk.

In this survey, we measured participants' lower extremity function using the SPPB, a structured, objective measurement for lower extremity physical performance, including standing balance, gait speed, and chair stands (Guralnik et al., 1994). More than one‐third of the patients on MHD had decreased lower extremity function, which supports previous research elucidating physical impairment in such patients. For example, Painter et al. (2016) showed that the average SPPB scores in patients on MHD were 9.4 ± 2.6, and similarities in the SPPB category distribution exist between this cohort and our study. Additionally, Roshanravan et al. reported that 31.7% of CKD patients (122/385) had worse timed up‐and‐go tests, and a 30% reduction in lower extremity performance was shown, compared to the normal predicted value (Painter et al., 2017).

Moreover, our research demonstrated that the FRS for CVD was correlated with the SPPB score and its components in patients on MHD. This is consistent with previous research findings that moderate SPPB scores were more closely associated with an increased CVD risk than high SPPB scores among older women, and low and very‐low SPPB scores demonstrated an even stronger association (Bellettiere et al., 2009). In a Japanese study, the prevalence of a knee extensor strength of less than 40% in patients on MHD was 47.4%, and a strong relationship between lower limb strength and mortality risk was reported (Roshanravan et al., 2013). Similarly, every additional 20 m of walking distance during the 6‐minute walking test could reduce all‐cause mortality by 12%, fatal and non–fatal cardiovascular events by 7%, and all‐cause hospitalization by 4% (Torino et al., 2014). Other studies have also revealed that poor performance in the SPPB and its components could predict a greater risk of all‐cause mortality in different populations (Cooper et al., 2010; Matsuzawa et al., 2014).

However, in contrast to lower extremity function, our data did not prove a significant correlation between handgrip power and CVD risk. While both upper and lower extremity functions objectively assess motor fitness as indices of physical performance, they may exert distinct effects on cardiovascular function and mortality. A systematic review concluded that handgrip strength is useful for assessing muscle function associated with nutritional status in patients on MHD (Leal et al., 2011). The European consensus on the definition and diagnosis of sarcopenia (EWGSOP2) has used handgrip strength for each test to assess evidence of sarcopenia (Cruz‐Jentoft et al., 2019). Moreover, older women with low handgrip strength had worse endothelial function, as assessed using the endothelial dysfunction (arterial health) test, thus supporting the association between the vascular system and sarcopenia (Yoo et al., 2018). Hence, low muscle strength, represented by low handgrip strength, has links to protein–energy wasting, physical inactivity, and mortality in patients on dialysis (Isoyama et al., 2014). The leading causes of low handgrip strength are a lack of physical activity and exercise due to vascular access of the upper extremity for hemodialysis. Furthermore, many other factors can influence handgrip measurements, such as equipment, body posture, and measurement position. In addition, lower extremity function can be associated with preload and cardiac output, organ perfusion, the renin–angiotensin–aldosterone system, and all related consequences, including CVD‐related and all‐cause mortalities (Halkar et al., 2020; Li et al., 2018; Srikanthan et al., 2016). In patients with heart failure, Cicoira et al. reported that the skeletal muscle mass of the legs could be associated with the peak oxygen consumption and poor survival, and may also be an independent predictor of future outcomes (Cicoira et al., 2001). A greater loss of quadriceps strength, rather than handgrip strength, was observed in healthy older people (Samuel et al., 2012), and improving walking pace appeared to reduce CVD risk (Welsh et al., 2020). Similarly, lower extremity performance in patients with CKD has shown a significant decline compared with that of handgrip strength, which was relatively preserved, influencing the 3‐year mortality more than other factors (Painter et al., 2017).

There are several potential explanations for the relative correlation between the SPPB score and CVD risk. The SPPB is a composite test based on muscle strength, physical activity, and balance (Penninx et al., 2000). Some studies have shown that the SPPB has a predictive value for mortality, hospitalization, nursing home admission, and disability (Guralnik et al., 1994, 1995; Matsuzawa et al., 2014; Penninx et al., 2000). Furthermore, the risk of all‐cause mortality increases when the SPPB score is <10 (Matsuzawa et al., 2014). The European guidelines on sarcopenia advise using the SPPB to assess the severity of sarcopenia because it predicts clinical outcomes (Cruz‐Jentoft et al., 2019; Reis et al., 2022). Sarcopenia involves the impairment of muscle strength and mass, and patients with the condition have increased all‐cause and CVD‐related mortalities in aged cohorts (Li et al., 2018; Zhang et al., 2018). Muscle wasting and subsequent fatty infiltration during sarcopenia could be associated with the higher CVD‐related and all‐cause mortalities owing to the poor exercise capacity and the physically inactive lives of patients with CVD (Srikanthan et al., 2016). In the frequent hemodialysis network trial, age, Black race, diabetes mellitus, peripheral arterial disease, intracellular water, and phase angle were inversely associated with SPPB scores (Kaysen et al., 2011). This is consistent with our finding that the CCI and the MIS significantly differed among the three SPPB groups.

The SPPB score, defined as physical performance associated with whole‐body function, can reflect the severity of sarcopenia and all‐cause mortality. In EWGSOP2, an SPPB score of 8 was the sarcopenia cutoff point for poor physical performance (Cruz‐Jentoft et al., 2019). In our study, an SPPB score less than 10 predicted a greater CVD risk, similar to the prediction value of the SPPB score for all‐cause mortality, as confirmed in previous literature (Matsuzawa et al., 2014). The SPPB shows great potential for evaluating physical function in the aged population and those with chronic diseases. The minimal detectable change score of the SPPB for 90% of the CIs was 1.7 points, which can represent meaningful changes in the physical performance of patients undergoing hemodialysis (Ortega‐Pérez et al., 2018). A physical intervention combining aerobic, strength, balance, and flexibility exercises can improve the SPPB score (LIFE Study Investigators et al., 2006). Based on these effects of exercise on SPPB scores, a study provides evidence that exercise specifically focusing on lower extremity function may reduce left ventricular mass and be safe, feasible, and well‐tolerated for patients on MHD (Graham‐Brown et al., 2021).

This study's results should be considered in the context of its strengths and limitations. This multicenter study was designed to evaluate the relationship between lower extremity functions and CVD risk in a relatively large number of patients undergoing hemodialysis in China. Furthermore, this study provides recommendations for interventions in nephrology rehabilitation programs to improve quality of life and prevent CVD in patients on hemodialysis. Moreover, it potentially reveals the different roles of upper and lower extremity functions in CVD risk. However, this study has some limitations. First, this cross‐sectional study may not account for the causality between physical performance and CVD risk. Second, this study excluded patients who underwent peritoneal dialysis and kidney transplant; therefore, some of our results on the upper limb may not be generalizable to those populations. Finally, all centers enrolled were in the region of Eastern China; however, to avoid bias, they were located in six different districts. Future studies with long‐term observation may provide strong and clear evidence of the causal relationship between physical performance and CKD. Interventional studies will also need to be conducted to validate effective protocols for the rehabilitation of patients on hemodialysis with poor lower extremity performance.

In conclusion, this study suggests that patients on hemodialysis may experience a decline in lower extremity function, which appears to be associated with an increased risk of CVD. This association remained significant even after adjusting for multiple factors. Additionally, the SPPB might serve as a useful tool for assessing physical performance for CVD risk in patients on hemodialysis. However, further studies are necessary to explore the longitudinal correlation between lower extremity function and CVD risk.

## AUTHOR CONTRIBUTIONS

Study concept and design: K.Z., X.L., C.Y., Q.G.; acquisition, analysis, and interpretation of data: C.Y., W.D., J.N., J.Z., L.Z., H.Q., S.Z.; drafting of the work: K.Z., X.L.; critical revision of the manuscript: QG, CY. All the authors contributed to the article and approved the submitted version.

## FUNDING INFORMATION

National Natural Science Foundation of China, Grant number: 82172552; Shanghai Sailing Program, Grant number: 22YF1417900; Clinical Research Project of Tongji Hospital of Tongji University. Grant number: ITJ(QN)2106 and ITJ(QN)2204.

## CONFLICT OF INTEREST STATEMENT

The authors declare no conflicts of interest.

## ETHICS STATEMENT

This study was conducted in accordance with the ethical standards of the Declaration of Helsinki. Ethical approval was obtained from the Ethics Committee of Tongji Hospital, Tongji University. The ethics approval number is K‐2020‐024.

## Data Availability

All data generated and analyzed are not available due to policy and ethical issues. Further inquiries can be directed to the corresponding author.
